# Detection of oncogenic mutations in resected bronchial margins by next-generation sequencing indicates early relapse in stage IA lung adenocarcinoma patients

**DOI:** 10.18632/oncotarget.16539

**Published:** 2017-03-24

**Authors:** Tangfeng Lv, Jiawei Zou, Hongbing Liu, Qin Shen, Zhenfeng Lu, XiaoJun Zhou, Xiaonan Wang, Yong Song

**Affiliations:** ^1^ Department of Respiratory Medicine, Jinling Hospital, Southern Medical University, Guangzhou, Nanjing, China; ^2^ Nanjing University Institute of Respiratory Medicine, Nanjing, China; ^3^ Department of Respiratory Medicine, Jinling Hospital, Nanjing University School of Medicine, Nanjing, China; ^4^ Department of Pathology, Jinling Hospital, Nanjing, China; ^5^ Department of Research and Development, Division of Precision Medicine, Geneseeq Technology Inc., Toronto, Ontario, Canada

**Keywords:** next-generation sequencing, early relapse, cancer-related genes, oncogene, surgical margins

## Abstract

Stage I non-small cell lung cancer (NSCLC) patients experience a relatively high rate of recurrence, ranging from about 30-35%. We hypothesized that this elevated risk of recurrence is due to the presence of tumor cells at bronchial margins which was undetected by conventional light microscopy. Patients with clinical stage IA (T1N0M0) NSCLC were enrolled in this study, which included 8 early-relapse(ER) and 6 no-relapse(NR) patients. Primary tumor, bronchial margin, and normal lung tissues were collected and sent to a central site for targeted next-generation sequencing analysis. All of the patients were lung adenocarcinoma. Gene mutations were identified in all tumor tissue samples (100%). Oncogenic mutations were identified in 87.5%(7/8) bronchial margins of early relapse patients, whereas only 16.7%(1/6) no-relapse (NR) patient of marginal tissue had identified gene mutation. Additionally, concordance between primary tumor and bronchial margins was relatively high, with 4 of 8 (50%) ER patients having at least one identical mutation. Moreover, according to the gene mutation status in marginal tissue, 87.5% (7/8) of patients with at least one gene mutation in the bronchial margins had local recurrence or metastasis, whereas only 16.7% (1/6) of patients without any mutation detected had signs of relapse, the recurrence rate was significantly higher than that of the negative mutation margin group ((*p* (log-rank) = 0.023). The existence of oncogenic mutations in bronchial margins may represent occult residual tumor and elevated risk of recurrence in early stage NSCLC patients. Thus, assessing molecular status in bronchial margins may help identify patients who might benefit from extensive surgery or adjuvant treatment.

## INTRODUCTION

Lung cancer is the leading cause of cancer-related death worldwide, and non-small cell lung cancer (NSCLC) accounts for 80-85% of all lung cancers [1, 2]. Surgery is the most effective treatment of NSCLC. Unfortunately, the 5-year survival rate of NSCLC patients is only about 30-60%, with local recurrence and metastasis being the main causes of the low survival rate. Although stage I NSCLC patients have a relatively better prognosis, they still experience a 30% risk of recurrence after a curative resection [3–5]. Current evidence recommends cisplatin-based chemotherapy as a standard treatment for stage II and III NSCLC patients after radical resection, and postoperative adjuvant chemotherapy may increase the 5-year survival rate by 4-15% in these patients [6, 7]. The majority of patients with stage IA and IB only undergo surgical resection.

Currently, surgical treatment is still the most effective treatment of stage I-IIIA NSCLC. Curative resection of NSCLC requires macroscopic and microscopic radical resection (R0-resection). However, about 5–15% of patients are left with microscopic (R1) or macroscopic (R2) residual disease at the surgical margin [8, 9]. The presence of residual disease at the surgical margin were associated with poor prognosis [8–12]. Hancock et al [11] identified 54512 NSCLC patients who underwent curative surgery between 2003 and 2006, of note, 5-year survival rate of stage pI patients with R1 margins decreased from 62% to 37% compared to patients with complete resection. Similarly, another study [10] also showed that patients with R1 margins have worse disease-free survival and 5-year survival. At present, the main method for confirming clean bronchial margins is still routine pathological diagnosis, however, roughly 20% of patients having no evidence of macroscopic or microscopic disease at the resected margins suffer from local recurrence after surgery [10].Conventional histologic evaluation may not provide sufficient information about the malignant potential of surgery margin. Thus, it is crucial to find a more sensitive and accurate means of examination to assess the surgical margins so that we can identify those patients with a high-risk of recurrence.

Previous studies have attempted to find molecularly malignant events in surgical margins that might identify those patients at high risk for developing recurrent disease, however, sometimes the results were controversial. By detecting mutations of the *k-ras* proto-oncogene, Masasyesva et al [13] found that high local recurrence rates of sublobar resection were associated with the occult presence of tumor cells at resection margins. Vatan et al [14] examined *p53* gene mutations and *k-ras* codon 12 mutations from the tumor samples and surgical margins of 34 NSCLC patients, and their mutation rate was found to be much lower than the range given in the literature. Moreover, they did not provide information about the prognosis of the patients. Guo et al [15] detected DNA promoter hypermethylation changes at the bronchial margins of primary tumors, in bronchoalveolar fluid, and in paired nonmalignant lung tissue, and they found that histologically negative bronchial margins of resected NSCLC exhibit frequent hypermethylation changes in multiple genes, but their data does not show any statistically significant correlation between the methylation status in the tumor or bronchial margin and local recurrence or distant metastases.

With the advancement of next-generation sequencing (NGS), it is now possible to identify oncogenic alterations that would have previously been missed by conventional pathological diagnosis. Rather than sequencing the entire genome or exome, the clinical cancer gene test, which includes genes that show frequent alterations in cancer, can reduce the amount of tissue, time, and effort required to perform sequencing. These panels use a PCR capture-based NGS assay that allows deep targeted sequencing of genes of interest from limited formalin-fixed, paraffin-embedded (FFPE) specimens [16], Thus, we attempt to test the hypothesis that the presence of occult residual tumor in the bronchial margins of stage I NSCLC would negatively affect both time to recurrence and survival after surgical resection.

## RESULTS

### Patient characteristics

**Figure 1:**
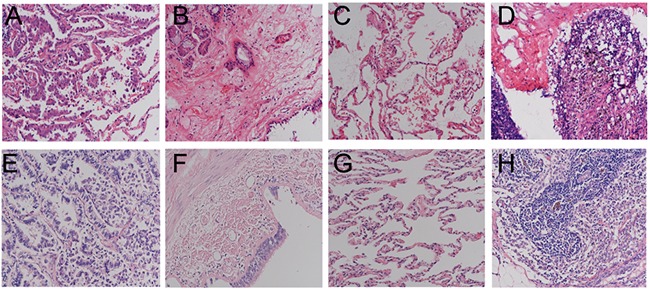
H&E staining pictures at 400× Magnification **(A)** tumor tissue of NR; **(B)** bronchia marginal tissue of NR; **(C)** normal lung tissue of NR; **(D)** lymph node tissue of NR; **(E)** tumor tissue of ER; **(F)** bronchia marginal tissue of ER; **(G)** normal lung tissue of ER; **(H)** lymph node tissue of ER. NR, no relapse; ER, early relapse.

**Figure 2:**
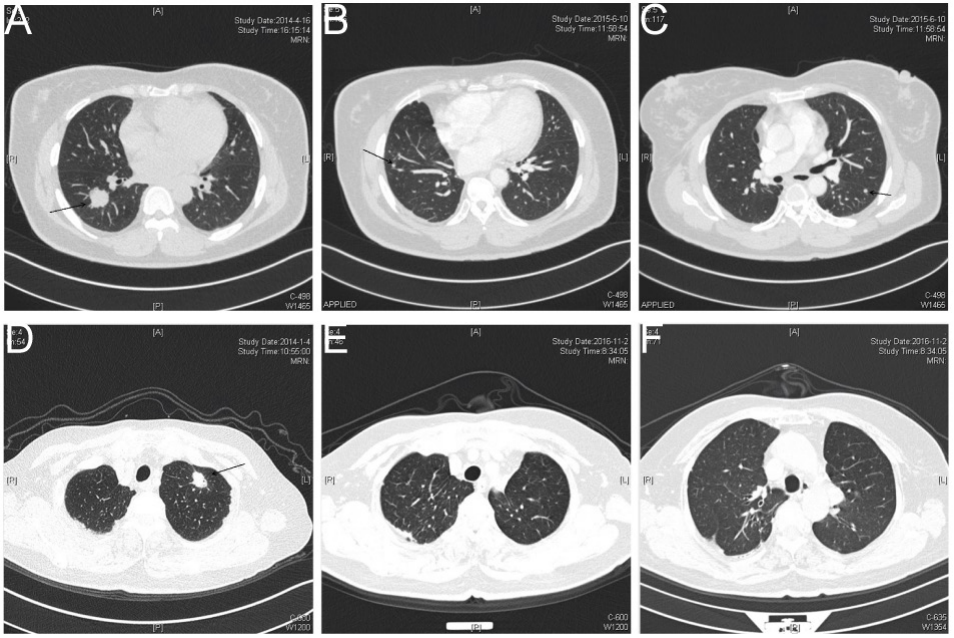
Chest computed tomography (CT) pictures before and after surgical resection Figure **(A)** chest CT picture for patient #4 in the ER group before surgical resection. Figure **(B)** and **(C)** chest CT picture for patient #4 in the ER group following postoperative recurrence. Figure **(D)** chest CT picture for patient #9 in the NR group before surgical resection. Figure **(E)** and **(F)** chest CT picture for patient #9 in the NR group who did not appear any signs of postoperative recurrence. The location of tumor node is indicated by arrows. NR, no relapse; ER, early relapse.

The clinical and pathologic characteristics of the enrolled patients are showed in Table 1. A total of 14 patients were enrolled, including 8 cases in the early relapse (ER) group and 6 cases in the no relapse (NR) group. The average age of the ER group was 61.37 ± 11.27 years old and the sex ratio (male (M)/female (F)) was 6:2; the average age of the NR group was 47.33 ± 14.38 years old and the sex ratio (M/F) was 3:3. There were 4 smokers (50%) in the ER group and 2 smokers (33.3%) in the NR group. The cancer type of patients in the ER group was mainly acinar predominant adenocarcinoma, while of the patients in the NR group, 3 cases were papillary predominant adenocarnoma, 2 cases were acinar predominant adenocarcinoma, and 1 was lepidic predominant adenocarnoma. Meanwhile, the TNM stage of all patients was IA, including 6 cases in the ER group and 3 cases in the NR group that were T1bN0M0. In terms of differentiation, 6 cases (75%) in the ER group were intermediate grade; in the NR group, 4 cases (66.7%) were intermediate grade. Meanwhile, no other tumor cell was identified in the bronchial margins or lymphoid tissue by microscopy (Figure 1). CT pictures of the chest before and after surgery are showed in Figure 2. The recurrence time of the ER group ranged from 4.13 to 22.7 months, with the average time being 12.72 months, whereas no local or systemic recurrence was identified in the NR group. Finally, the follow up time of the ER group ranged from 7.4 to 38.5 months, with the average time being 24.4 ± 11.9 months, while the follow up time of the NR group ranged from 32.7 to 54.1, with the average time being 41.4 ± 8.1 months. Site of recurrences in ER group were shown in Table 2.

**Table 1:** Patient characteristics

Characteristics	Patients ER (n=8)	Patients NR (n=6)
**Median age, years (range)**	61.37 ± 11.27 (46-77)	47.33 ± 14.38(32-66)
**Median follow-up, months (range)**	24.4 ± 11.9 (7.4-38.5)	41.4 ± 8.1 (32.7-54.1)
**Gender: Male/Female (%)**	6/2 (75%/25%)	3/3 (50%/50%)
**Ever smokers: Yes/No (%)**	4/4 (50%/50%)	2/4 (33.3%/66.7%)
**Histologic subtype**		
Acinar/Papillary/Lepidic (%)	8/0/0 (100%/0%/0%)	2/3/1(33.3%/50% %/16.7%)
**TNM stage**		
T1aN0M0/T1bN0M0	2/6 (25%/75%)	3/3 (50%/50%)
**IA (%)**	8 (100%)	6 (100%)
**operation selection**		
segmentectomy	8 (100%)	6 (100%)
**Differentiation**		
High/Intermediate/low	1/6/1 (12.5%/75%/12.5%)	2/4/0 (33.3%/66.7%/0%)

Abbreviations: early relapse (ER); no relapse (NR)

**Table 2:** Site of recurrences in ER group

Patients (n=8)	site of recurrences
**NO 1**	Chest CT showed multiple metastasis in the both site of lungs, lung adenocarcinoma were found in one of right pulmonary nodule by CT-PTNB.
**NO 2**	PET-CT showed multiple metastasis in the right lung
**NO 3**	Chest/ Abdominal CT showed multiple metastases in the right lung, left pleural, thoracic vertebral and liver.
**NO 4**	Chest CT showed multiple metastasis in the both site of lungs.
**NO 5**	Chest CT showed multiple metastasis in the both site of lungs, tumor cells were found in the right pleural effusion.
**NO 6**	PET-CT showed metastasis in left chest wall and surrounding rib invasion, lung adenocarcinoma were found in chest wall by CT-PTNB.
**NO 7**	Chest CT showed multiple metastasis in the right pleural, and one round nodules in right lower lobe
**NO 8**	Chest CT showed one round nodules in right lower lobe, with mediastinal and hilar lymphadenopathy

Abbreviations: early relapse (ER); computed tomography-guided percutaneous transthoracic needle biopsy (CT-PTNB). computed tomography(CT)

### Investigation of gene mutations in tumor and marginal tissue between ER and NR groups

**Figure 3:**
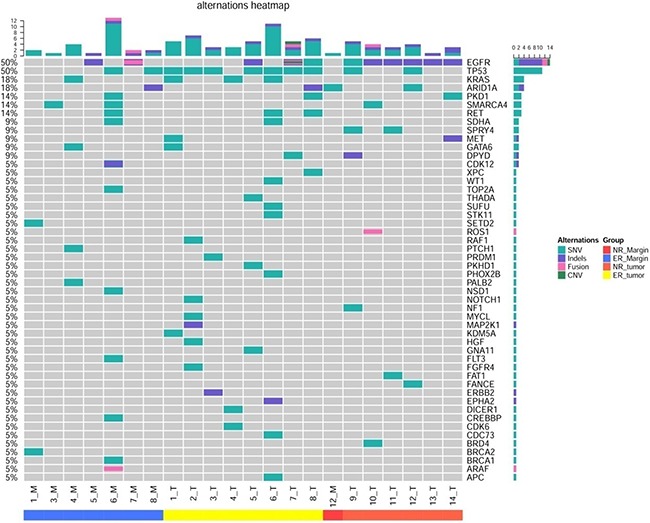
Heat map for genes X axis represents the tissue type and number of patient The samples from left to right are ER marginal tissue, ER tumor tissue, NR marginal tissue, and NR tissue. M, marginal tissue; T, tumor tissue; ER, early relapse; NR, no relapse

Among the ER group, gene mutations were detected in all of the tumor tissue samples. The number of mutations ranged from 3 to 11, with the average being 5.875. Gene mutations were identified in 7 cases (87.5%) of the marginal tissue (Table 3). The mutation number ranged from 1 to 16, with the average being 3.5. *TP*53 was the most common mutation identified in the tumor tissue of ER group, accounting for 62.5% (5/8) of the patients; *K-ras* and *EGFR* mutations were the second most common, accounting for 37.5% (3/8) of the patients.(Figure 3 and Supplementary Table 1). Among the 3 patients with *EGFR* mutation, one had a *19-Del* mutation, one had a *19-Del* mutation and *PSH-EGFR* fusion, and one had a *L858R* mutation. Moreover, mutations of *EGFR*, *TP53*, and *SMARCA4* were the common mutations identified in marginal tissue of ER group, and the detection rates were all 28.6% (2/7) (Figure 3 and Supplementary Table 1).. Between the tumor and marginal tissues of ER group, the key mutations in 50% of the cases (#4, #5, #7 and #8) were the same (Table 3 and Figure 3). Among the NR group, gene mutations were identified in all 6 tumor tissue samples (100%). The number of mutations ranged from 1 to 8 and the average number was 3.83. However, only 1 sample (16.7%) of marginal tissue had an identified gene mutation and this was a single mutation (Table 4). The *EGFR* mutation was the most common mutation detected in the tumor tissue of NR group and was identified in all of the 6 cases (100%), including 3 cases of *EGFR 19-Del*, 1 case of a co-mutation of *EGFR L858R* and *EGFR V689V*, 1 case of *EGFR 746-756 Del* and *ZNF385D-ROS1* fusion, and 1 case of *EGFR M766 delins MASV*.(Table 4 and Figure 3) The unique mutation in the marginal tissue in the NR group was identified in patient #12. Additionally, 4 mutations were also discovered in tumor tissue of patient #12, with *EGFR 19Del* and *TP53 P152L* having the highest abundance. Although another mutation *ARID1A* was discovered in tumor tissue, no key mutation, such as *EGFR* or *TP53*, was identified in corresponding marginal tissue (Figure 3 and Table 4). Detailed information regarding the patients is provided in Supplementary Table 1. *EGFR* Mutation of Tumors tissue were also tested by amplification-refractory mutation system (ARMS). All of patient underwent EGFR mutation test. Of note, *EGFR* (n=6) mutations were detected by conventional sequencing. However, *EGFR 19-del* was exclusively detected in the tumor tissue of NO12 patient by NGS, while standard molecular testing (ARMS) is negative. There were totally 5 of *EGFR* mutations not previously covered by the amplification-refractory mutation system (NO7. *PSPH-EGFR* *Fusion*, NO9. *EGFR V689V*, NO10. *EGFR 746_750del*, NO11. *EGFR M766delinsMASV*, NO12. *EGFR 19del*). Detailed information regarding the patients is provided in Supplementary Table 2

**Table 3:** Mutations in patients with early relapse

Patients (n=8)	Tumor			Margin Tissue		
	Total number Of mutations	Key Mutations	MAF (%)	Total number of mutations	Key Mutations	MAF (%)
**NO 1**	5	KRAS G12DTP53 P47L	21.4%1.3%	2	None detected	-
**NO 2**	7	TP53 V172F TP53 deletion	22.5%	0	None detected	-
**NO 3**	3	ERBB2 771insAYVMTP53 E294X	21.1%13.2%	1	None detected	-
**NO 4**	3	KRAS D57N	1.2%	4	KRAS D57N	0.5%
**NO 5**	5	EGFR 19del	10.8%	1	EGFR 19del	2.8%
**NO 6**	11	KRAS G12CTP53 R158L	23.1%25.5%	16	None detected	-
**NO 7**	5	EGFR 19delPSPH-EGFR Fusion	59.9%9.7%	2	EGFR 19delPSPH-EGFR Fusion	3.0%1.1%
**NO 8**	8	EGFR L858RTP53 Y220C	37.4%39.1%	2	TP53 Y220C	1.0%

**Table 4:** Mutations in patients without recurrence

Patients(n=6)	Tumor			Margin Tissue		
	Total number Of mutations	Key Mutations	MAF (%)	Total number Of mutations	Key Mutations	MAF (%)
**NO9**	8	EGFR L858REGFR V689VTP53 S127T	34.1%32.1%46.8%	0	Not detected	-
**NO10**	4	EGFR 746_750delZNF385D-ROS1 Fusion	12.9%7.1%	0	Not detected	-
**NO11**	3	EGFR M766delinsMASV	15.6%	0	Not detected	-
**NO12**	4	EGFR 19delTP53 P152L	50.4%60.4%	1	Not detected	-
**NO13**	1	EGFR 19del	61.5%	0	Not detected	-
**NO14**	3	EGFR 19del	5.4%	0	Not detected	-

Abbreviations: MAF: Minor Allele Frequency.

### Correlation between gene mutation in marginal tissues and recurrence

**Figure 4:**
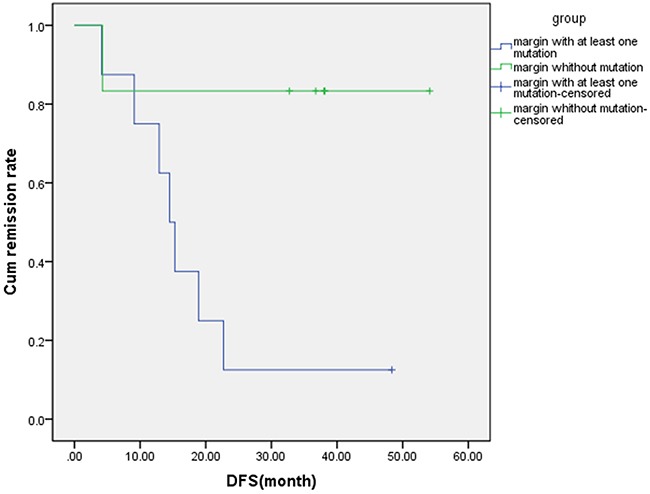
The relationship between number of mutation genes in margin tissue and disease occurrence Based on patient characteristics, 8 patients were enrolled in the margin with at least one mutation group and 7 of them have palindromia. Meanwhile, 6 patients were enrolled in the margin without mutation and 1 of them has palindromia. DFS, disease free survival.

Based on the results of high-throughput sequencing, 7 out of 8 (87.5%) tumor specific gene mutations were identified in the marginal tissue in the ER group, while genetic mutation in the marginal tissue was only observed in 1 out of 6 patients (12.5%) in the NR group. After analysis by Fisher's exact test, a significant difference was identified between these two groups (p =0.016) (Table 5). Moreover, according to the mutation status in marginal tissues, all of the enrolled patients were divided into two group: a) with at least one mutation (8 cases), and b) without mutation (6 cases). Subsequently, the results of the follow up analysis were evaluated. The follow up time of the group with at least one mutation ranged from 4.13 to 48.36 months, with the average being 18.23 months. The follow up time for the group without gene mutation ranged from 4.27 to 54.13 months, the average being 34.0 months. In addition, palindromia occurred in 7 out of 8 patients (87.5%) in the group with at least one mutation. However, only one (16.7%) palindromia was identified in the group without mutation, which was significantly lower than that of the group with at least one mutation (*p* (log-rank) = 0.023) (Figure 4).

**Table 5:** Comparison of positive surgical margins between ER and NR groups

group	Detected any mutation in margin	Without mutation in margin	P Value*
NR	1	5	0.016
ER	7	1	

Abbreviations: early relapse (ER); no relapse (NR)

*Fisher's exact test, 2-tailed

## DISCUSSION

Local recurrence or metastasis is generally believed to account for the failure of therapy and relapse after initial surgical resection [17], despite histological confirmation of tumor-free surgical margins. Additionally, the molecular status of the “normal” appearing lung tissue in the surgical margins and its clinical significance has rarely been examined in NSCLC. Although Stage I NSCLC patients have better a prognosis, with 5-year survival rates ranging from 40% to 90%, among the patients with stage I disease, nearly 30-35% of them will relapse [3, 5, 18]. If we can identify early-stage NSCLC patients with a great chance of relapse, we could select the appropriate candidate treatment such as adjuvant therapy or closer follow-up. We hypothesized that occult tumor cells at bronchial margins could be detected in patients with no histological evidence of malignancy, and they may lead to a high risk of early relapse in radically resected lung adenocarcinoma patients. Compared to NR patients, oncogene mutations were detected more frequently in resected bronchial margins of ER patients. In addition, concordance between primary tumor and bronchial margins was relatively high, with 4 of 8 (50%) of patients having at least one identical mutation. The results also showed that 87.5% (7/8) of patients with at least one gene mutation in bronchial margins had local recurrence or metastasis. In contrast, only 16.7% (1/6) of patients who did not have any detected mutations in their bronchial margins had signs of relapse. The recurrence rate was significantly higher in the group with detected gene mutations than that of the negative molecular margin group (*p*(log-rank)=0.023). We assume that those oncogenic changes in bronchial margins represent occult residual tumor, and detection of these changes can help identify patients with a high risk for relapse after resection. Such patients may benefit from more extensive surgery or postoperative chemotherapy, which may help improve their overall survival.

Given the unsatisfactory survival rates, many researchers have been searching for possible methods that could help predict the outcome of stage I patients, such as analysis of the standard uptake value (SUVmax) in positron emission tomography or carcinoembryonic antigen (CEA) serum levels [19, 20], or detection of genetic alterations [21–23], epigenetic modifications [15, 24], and gene-expression profiles [25–27]. However, few studies have explored the molecular status of histologically normal-appearing surgical margins [13–15, 21, 28]. Masasyesva et al [13] focused on the molecular margins of sublobar resections of lung cancer by analysis of surgical margins obtained from pulmonary wedge resections for stage I and II lung cancer. Despite histologically tumor-free surgical margins, they found that k-ras mutant cells could be detected in these surgical margins and that the presence of these cells was associated with a higher incidence of local recurrence and disease progression. However, their study is limited to patients with k-ras mutant tumors and is not widely applicable to all NSCLCs. Now, using NGS, the addition of mutational analysis for other genes would give us a more comprehensive genetic landscape and allow application to the majority of NSCLCs. Guo et al [15] detected DNA promoter hypermethylation changes in bronchial margins, primary tumors, bronchoalveolar fluid, and paired nonmalignant lung tissue, which were obtained from 20 NSCLC patients who underwent surgery. They did not find any statistically significant correlation between the methylation status of resected bronchial margin and regional recurrence or distant metastases. However, they did find that recurrence only occurred in patients with methylation-positive bronchial margins. Vatan et al [14] examined p53 gene mutations and *k-ras* codon 12 mutations from tumor samples and surgical margins of 34 NSCLC patients. Their mutation rate was found to be much lower than the range given in the literature. In addition, they did not provide any information about the prognosis of the patients.

In a recent study, Cao et al [28] explored the molecular status of the negative surgical margins (NSMs) of 60 lung squamous cell carcinoma patients using microarrays and validated quantitative RT-PCR. They concluded that the epithelial-to-mesenchymal (EMT)-like gene-expression subtype identified in NSMs was associated with lymph node metastasis and poor overall survival of NSCLC patients. Our results differ in some aspects. Firstly, Cao et al mainly investigated the prognostic relevance of EMT molecular events in NSMs in patients with NSCLC. Secondly, the study population of their research included patients with stage II/III lung cancer, whereas our study included exclusively patients with stages IA (T1N0M0) lung adenocarcinoma where the tumor lesion was less than 3 cm. Thirdly, their histologic subtypes mainly consisted of lung squamous cell carcinoma, whereas all of the histologic subtypes in our study were lung adenocarcinoma. Fourth, they confirmed the prognostic value of four EMT-related genes in NSMs, whereas our study determined that mutations were mainly detected in resected bronchial margins of ER patients. We suppose these gene mutation events may represent minimal residual disease, and after a critical threshold of events, these change may lead to rapid tumor progression.

In sum, we can conclude that the detection of oncogenes in microscopically negative bronchial margins increased the risk of relapse in stage I lung adenocarcinoma patients. Thus, we propose that assessment of microscopically negative bronchial margins by NGS can serve as a potential “molecular margin”, and may be helpful in predicting outcome of NSCLC patients. However, our study also has several limitations. Firstly, our study sample number was relatively small. Secondly, we did not have any information regarding local-regional recurrence because most of patients did not undergo postoperative bronchoscopy examination. Further studies are needed to confirm the clinical impact of the detection of oncogenes in bronchial margins, and if in the future NGS might be of useful in identifying stage I patients with a worse prognosis who may benefit from adjuvant treatment.

## MATERIALS AND METHODS

### Patient enrollment and sample preparation

We identified 205 patients with stage I disease (TNM version 7.0) from a series of 653 patients who underwent radically surgical resection from Nanjing general hospital between December 2010 and May 2015. Exclusion criteria included: a) patients who were not diagnosed pathologically as stage I lung cancer; b) patients with positive resected bronchial margins by pathology; c) patients without follow-up information of recurrence and overall survival; and d) patients that had evidence of distant metastasis before surgery. We defined NR as patients without evidence of relapse, median follow-up time of 41 months (range 32.7-54.1 months). ER were those patients who relapsed within two years, median follow-up time of 24 months (range 7.4-38.5 months). Finally, a total of 14 patients were enrolled in the study, 8 in the ER group and 6 in the NR group.

We examined primary tumor, bronchial margin, and normal lung tissues. All patients had no evidence of macroscopic or microscopic disease at the resected bronchial margins (Figure 1). Normal lung tissue taken from distant lung areas from patients with adenocarcinoma was used as calibrator for microarray and quantitative PCR (Q-PCR) analysis. For FFPE tumor samples, only samples with a tumor cell content above 20% were considered eligible and included in the study. This study design was approved by the Ethics Committee of Nanjing General Hospital, who waived the need for informed consent due to the non-invasive nature of the study and because of patient anonymity. Collected samples were sent to the core facility of Nanjing Shihe Jiyin Biotechnology Inc. (Nanjing, China) for targeted NGS analysis.

### DNA extraction

Five to eight 10 μm tissue sections from tumor, margin, and normal lung tissues were obtained. FFPE samples were used for genomic DNA extraction with the QIAamp DNA FFPE Tissue Kit (QIAGEN) following the manufacturer's instructions. Genomic DNA of cellular sediments of pleural effusions were prepared with the DNeasy Blood & Tissue kit (QIAGEN). Normal tissue DNA was sequenced together with tumor and margin DNA samples for the purpose of identifying germline mutations. DNA quality was assessed using a Nanodrop2000 (Thermo Fisher Scientific) and the quantity was measured using the dsDNA HS Assay Kit (Life Technologies) on Qubit 2.0.

### Library preparation and sequencing

Extracted tumor genomic DNA was fragmented into 300~350 bp fragments using a Covaris M220 instrument (Covaris). Sequencing libraries were prepared with the KAPA Hyper Prep kit (KAPA Biosystems) with optimized protocols. In brief, sheared tissue DNA was subjected to end-repairing, A-tailing, adapter ligation, and size selection using Agencourt AMPure XP beads (Beckman Coulter). Libraries were then subjected to PCR amplification and purification before targeted enrichment.

DNA libraries from different samples were marked with unique indices during library preparation and up to 2 μg of different libraries were pooled together for targeted enrichment. Human cot-1 DNA (Life Technologies) and xGen Universal blocking oligos (Integrated DNA Technologies) were added to block nonspecific binding of library DNA to targeted probes. Customized xGen lockdown probe panels (Integrated DNA Technologies) were used for targeted enrichment of 416 predefined genes. The hybridization reaction was performed by using the NimbleGen SeqCap EZ Hybridization and Wash Kit (Roche). Dynabeads M-270 (Life Technologies) was used to capture probe-bind fragments, followed by library amplification with Illumina p5 (5’ AAT GAT ACG GCG ACC GA 3’) and p7 primers (5’ CAA GCA GAA GAC GGC ATA CGA GAT 3’) in KAPA HiFi HotStart ReadyMix (KAPA Biosystems), and purification by Agencourt AMPure XP beads. Library quantification was analyzed using the KAPA Library Quantification kit (KAPA Biosystems). The size distribution of libraries was measured using the Agilent Technologies 2100 Bioanalyzer (Agilent Technologies). The enriched libraries were sequenced on Hiseq 4000 NGS platforms (Illumina) to coverage depths of at least 100x and 300x after removing PCR duplicates for normal FFPE and tumor/margin FFPE, respectively.

### EGFR mutation analysis tested by conventional methods

DNA was extracted from five pieces of formalin-fixed, paraffin-embedded(FFPE) tumor tissue using the QIAamp FFPE Tissue Kit. Molecular analysis of mutation status of EGFR exons 18, 19, 20, and 21 was examined with Human EGFR Gene Mutations Detection Kit (AmoyDx, Xiamen, China), which is a polymerase chain reaction (PCR)–based amplification-refractory mutation system (ARMS).

### Statistical analysis

Categorical variables were analyzed by chi-square tests, except where a small sample size (<5) required the use of Fisher's exact test. Remission rate was estimated using the Kaplan-Meier method and is presented as a median value with a two-sided 95% confidence interval (CI). A two-sided log-rank test was used to compare the remission rates between the two groups. All reported *p*-values were two-tailed, and *p*-values less than 0.05 were considered statistically significant. Statistical analyses of the data were performed using SPSS version 21 (SPSS Inc., Chicago, IL).

## SUPPLEMENTARY MATERIALS TABLES




