# Supplementary material for: Detection of oncogenic mutations in resected bronchial margins by next-generation sequencing indicates early relapse in stage IA lung adenocarcinoma patients
Source: Oncotarget. 2017 Mar 24;8(25):40643–53. doi: 10.18632/oncotarget.16539 (PMC5522272; doi:10.18632/oncotarget.16539)
Supplement: Supplementary file 1 [file oncotarget-08-40643-s001.pdf]

Detection of oncogenic mutations in resected bronchial margins by next-generation sequencing indicates early relapse in stage IA lung adenocarcinoma patients

SUPPLEMENTARY TABLES

Supplementary Table 1: Mutation information of all patients

See Supplementary File 1

Supplementary Table 2: *EGFR* mutation information of tumors tissue tested by conventional methods

Patient	Gene	Exons	AA change
NO1	-	-	-
NO2	-	-	-
NO3	-	-	-
NO4	-	-	-
NO5	*EGFR*	19	p.746_750del (c.2236_2250delGAATTAAGAGAAGCA)
NO6	-	-	-
NO7	*EGFR*	19	p.746_750del (c.2236_2250delGAATTAAGAGAAGCA)-
NO8	*EGFR*	21	p.L858R (c.T2573G)
NO9	*EGFR*	21	p.L858R (c.T2573G)
NO10	-	-	-
NO11	-	-	-
NO12	-	-	-
NO13	*EGFR*	19	p.746_750del (c.2236_2250delGAATTAAGAGAAGCA)
NO14	*EGFR*	19	p.746_750del (c.2236_2250delGAATTAAGAGAAGCA)

EGFR Mutation were detected by amplification-refractory mutation system (ARMS).
