# Supplementary material for: Detection of oncogenic mutations in resected bronchial margins by next-generation sequencing indicates early relapse in stage IA lung adenocarcinoma patients
Source: Oncotarget. 2017 Mar 24;8(25):40643–53. doi: 10.18632/oncotarget.16539 (PMC5522272; doi:10.18632/oncotarget.16539)
Supplement: Supplementary file 2 [file oncotarget-08-40643-s002.docx]

**Supplementary Table 1 Mutation information of all patients**

| **Patient** | **Gene** | **Exons** | **AA change** | **Chr start** | **Ref** | **Alt** | **MAF** |
| --- | --- | --- | --- | --- | --- | --- | --- |
| **NO1**  **Tumor** | *GATA6* | 2 | p.G104D (c.G311A) | chr18:19751416 | G | A | 1.74% |
|  | *KDM5A* | 14 | p.N600S (c.A1799G) | chr12:438170 | T | C | 10.33% |
|  | *KRAS* | 2 | p.G12D (c.G35A) | chr12:25398284 | C | T | 21.36% |
|  | *MET* | 20 | p.G1298E (c.G3893A) | chr7:116435749 | G | A | 19.70% |
|  | *TP53* | 4 | p.P47L (c.C140T) | chr17:7579547 | G | A | 1.26% |
| **NO1**  **Margin** | *BRCA2* | 10 | p.P389L (c.C1166T) | chr13:32906781 | C | T | 2.17% |
|  | *SETD2* | 3 | p.R329Q (c.G986A) | chr3:47165140 | C | T | 1.83% |
|  |  |  |  |  |  |  |  |
| **NO2**  **Tumor** | *FGFR4* | 15 | p.P652T (c.C1954A) | chr5:176523297 | C | A | 20.30% |
|  | *HGF* | 10 | p.D416H (c.G1246C) | chr7:81350086 | C | G | 33.72% |
|  | *MAP2K1* | 3 | p.101_103del (c.302_307delTGGAGA) | chr15:66729094 | TGGAGA | - | 18.65% |
|  | *MYCL* | 2 | p.D194N (c.G580A) | chr1:40366617 | C | T | 16.82% |
|  | *NOTCH1* | 34 | p.H2500D (c.C7498G) | chr9:139390693 | G | C | 31.91% |
|  | *RAF1* | 11 | p.R391K (c.G1172A) | chr3:12633228 | C | T | 20.63% |
|  | *TP53* | 5 | p.V172F (c.G514T) | chr17:7578416 | C | A | 22.46% |
| **NO2**  **Margin** | *-* |  |  |  |  |  |  |
|  |  |  |  |  |  |  |  |
| **NO3**  **Tumor** | *ERBB2* | 20 | p.771insAYVM (c.2310_2311insGCATACGTGATG) | chr17:37880981 | - | GCATACGTGATG | 23.13% |
|  | *PRDM1* | 6 | p.A616T (c.G1846A) | chr6:106554318 | G | A | 1.34% |
|  | *TP53* | 8 | p.E294X (c.G880T) | chr17:7577058 | C | A | 13.17% |
| **NO3**  **Margin** | *SMARCA4* | 7 | p.R377H (c.G1130A) | chr19:11100004 | G | A | 1.38% |
|  |  |  |  |  |  |  |  |
| **NO4**  **Tumor** | *DICER1* | 23 | p.R1599Q (c.G4796A) | chr14:95562461 | C | T | 1.80% |
|  | *CDK6* | 4 | p.R131X (c.C391T) | chr7:92355086 | G | A | 1.97% |
|  | *KRAS* | 3 | p.D57N | chr12:25,380,222-25,380,356 | C | T | 1.15% |
| **NO4**  **Margin** | *PALB2* | 5 | p.E837K (c.G2509A) | chr16:23640966 | C | T | 2.17% |
|  | *GATA6* | 7 | p.V572I (c.G1714A) | chr18:19780712 | G | A | 1.03% |
|  | *PTCH1* | 23 | p.E1428K (c.G4282A) | chr9:98209256 | C | T | 1.64% |
|  | *KRAS* | 3 | p.D57N | chr12:25,380,222-25,380,356 | C | T | 0.46% |
|  |  |  |  |  |  |  |  |
| **NO5**  **Tumor** | *EGFR* | 19 | p.746_750del (c.2236_2250delGAATTAAGAGAAGCA) | chr7:55242466 | GAATTAAGAGAAGCA | - | 10.78% |
|  | *GNA11* | 7 | p.A304V (c.C911T) | chr19:3121008 | C | T | 4.33% |
|  | *PKHD1* | 64 | p.S3823L (c.C11468T) | chr6:51503685 | G | A | 3.67% |
|  | *THADA* | 29 | p.H1356Y (c.C4066T) | chr2:43625271 | G | A | 10.62% |
|  | *TP53* | 8 | p.R282W (c.C844T) | chr17:7577094 | G | A | 11.56% |
| **NO5**  **Margin** | *EGFR* | 19 | p.746_750del (c.2236_2250delGAATTAAGAGAAGCA) | chr7:55242466 | GAATTAAGAGAAGCA | - | 2.82% |
|  |  |  |  |  |  |  |  |
| **NO6**  **Tumor** | *RET* | 3 | p.R114H (c.G341A) | chr10:43597793 | G | A | 53.90% |
|  | *SDHA* | 6 | p.I247V (c.A739G) | chr5:228417 | A | G | 38.00% |
|  | *PHOX2B* | 3 | p.K168R (c.A503G) | chr4:41748266 | T | C | 32.10% |
|  | *APC* | 2 | p.N32S (c.A95G) | chr5:112090682 | A | G | 3.51% |
|  | *CDC73* | 6 | p.E158K (c.G472A) | chr1:193107263 | G | A | 32.00% |
|  | *EPHA2* | 10 | p.618_619del (c.1854_1856delGAT) | chr1:16459984 | ATC | - | 1.78% |
|  | *KRAS* | 2 | p.G12C (c.G34T) | chr12:25398285 | C | A | 23.12% |
|  | *STK11* | 1 | p.K48X (c.A142T) | chr19:1207054 | A | T | 27.63% |
|  | *SUFU* | 7 | p.K303I (c.A908T) | chr10:104357048 | A | T | 18.72% |
|  | *TP53* | 5 | p.R158L (c.G473T) | chr17:7578457 | C | A | 25.49% |
|  | *WT1* | 1 | p.K3N (c.G9T) | chr11:32452077 | C | A | 11.17% |
| **NO6**  **Margin** | *RET* | 3 | p.R114H (c.G341A) | chr10:43597793 | G | A | 23.08% |
|  | *SDHA* | 6 | p.I247V (c.A739G) | chr5:228417 | A | G | 17.58% |
|  | *PKD1* | 16 | p.A2312V (c.C6935T) | chr16:2158014 | G | A | 15.85% |
|  | *NSD1* | 5 | p.R1159Q (c.G3476A) | chr5:176638876 | G | A | 15.09% |
|  | *SMARCA4* | 4 | p.Q201L (c.A602T) | chr19:11097111 | A | T | 10.22% |
|  | *SDHA* | 2 | p.V37I (c.G109A) | chr5:223642 | G | A | 9.34% |
|  | *FLT3* | 24 | p.P986S (c.C2956T) | chr13:28578215 | G | A | 19.63% |
|  | *TOP2A* | 8 | p.F267V (c.T799G) | chr17:38568061 | A | C | 14.50% |
|  | *PKD1* | 38 | p.3695_3713del (c.11083_11139delCATGGGCACGCCTACCGTCTGCAAAGCGCCATCAAGCAGGAGCTGCACAGCCGGGCC) | chr16:2142972 | GGCCCGGCTGTGCAGCTCCTGCTTGATGGCGCTTTGCAGACGGTAGGCGTGCCCATG | - | 11.74% |
|  | *TP53* | 8 | p.E294X (c.G880T) | chr17:7577058 | C | A | 8.38% |
|  | *CDK12* | 1 | p.D103Y (c.G307T) | chr17:37618631 | G | T | 5.02% |
|  | *SMARCA4* | 34 | p.R1591Q (c.G4772A) | chr19:11170724 | G | A | 4.25% |
|  | *CDK12* | 1 | p.343_344del (c.1028_1030delGTC) | chr17:37619352 | GTC | - | 3.76% |
|  | *BRCA1* | 10 | p.Y856H (c.T2566C) | chr17:41244982 | A | G | 12.33% |
|  | *CREBBP* | 2 | p.K266T (c.A797C) | chr16:3900299 | T | G | 20.37% |
|  | *SSX9-ARAF* | SSX9:E5-ARAF:I8 | Fusion | chrX:48161012 |  |  | 18.00% |
|  |  |  |  |  |  |  |  |
| **NO7**  **Tumor** | *DPYD* | 17 | p.G727C (c.G2179T) | chr1:97771733 | C | A | 2.96% |
|  | *EGFR* | 19 | p.746_750del (c.2236_2250delGAATTAAGAGAAGCA) | chr7:55242466 | GAATTAAGAGAAGCA | - | 59.92% |
|  | *TP53* | 8 | p.R273L (c.G818T) | chr17:7577120 | C | A | 23.62% |
|  | *EGFR* |  | Amplify 3.25 fold |  |  |  |  |
|  | *PSPH-EGFR* | PSPH：I17-EGFR:E28 | Fusion | chr7:55,272,900-55,273,222 |  |  | 9.70% |
| **NO7**  **Margin** | *EGFR* | 19 | p.746_750del (c.2236_2250delGAATTAAGAGAAGCA) | chr7:55242466 | GAATTAAGAGAAGCA | - | 2.96% |
|  | *PSPH-EGFR* | PSPH：I17-EGFR:E28 | Fusion | chr7:55,272,900-55,273,222 |  |  | 1.10% |
|  |  |  |  |  |  |  |  |
| **NO8**  **Tumor** | *AKT1* | 12 | p.R370H (c.G1109A) | chr14:105239278 | C | T | 1.16% |
|  | *ARID1A* | 1 | p.G84delinsGGGGAGS (c.250_251insGCGGCGGCGGAGCCGGCA) | chr1:27023144 | - | GCGGCGGCGGAGCCGGCA | 15.32% |
|  | *EGFR* | 21 | p.L858R (c.T2573G) | chr7:55259515 | T | G | 37.37% |
|  | *PKD1* | 15 | p.T1432M (c.C4295T) | chr16:2160873 | G | A | 1.85% |
|  | *PKD1* | 18 | p.T2409M (c.C7226T) | chr16:2156662 | G | A | 1.31% |
|  | *RET* | 6 | p.S401N (c.G1202A) | chr10:43604617 | G | A | 2.35% |
|  | *TP53* | 6 | p.Y220C (c.A659G) | chr17:7578190 | T | C | 39.05% |
|  | *XPC* | 13 | p.V791I (c.G2371A) | chr3:14190111 | C | T | 3.42% |
| **NO8**  **Margin** | *TP53* | 6 | p.Y220C (c.A659G) | chr17:7578190 | T | C | 1.00% |
|  | *ARID1A* | 1 | p.G84delinsGGGGAGS (c.250_251insGCGGCGGCGGAGCCGGCA) | chr1:27023144 | - | GCGGCGGCGGAGCCGGCA | 9.52% |
|  | | | | | | | |
| **NO9**  **Tumor** | *DPYD* | 11 | p.V404fs (c.1212_1224delTGCTATGCAGTTT) | chr1:98039431 | AAACTGCATAGCA | - | 15.68% |
|  | *EGFR* | 21 | p.L858R (c.T2573G) | chr7:55259515 | T | G | 34.13% |
|  | *EGFR* | 18 | p.V689L (c.G2065C) | chr7:55241617 | G | C | 32.14% |
|  | *NF1* | 33 | p.R1462W (c.C4384T) | chr17:29586101 | C | T | 1.35% |
|  | *SPRY4* | 3 | p.V171I (c.G511A) | chr5:141694232 | C | T | 1.62% |
|  | *TP53* | 5 | p.S127T (c.T379A) | chr17:7578551 | A | T | 46.76% |
| **NO9**  **Margin** | *-* |  |  |  |  |  |  |
|  |  |  |  |  |  |  |  |
| **NO10**  **Tumor** | *EGFR* | 19 | p.746_750del (c.2236_2250delGAATTAAGAGAAGCA) | chr7:55242466 | GAATTAAGAGAAGCA | - | 12.90% |
|  | *BRD4* | 2 | p.T60A (c.A178G) | chr19:15383733 | T | C | 6.91% |
|  | *SMARCA4* | 34 | p.R1613Q (c.G4838A) | chr19:11170790 | G | A | 1.54% |
|  | *ZNF385D-ROS1* | ZNF385D：I4-ROS1：I34 | Fusion | chr3:21522931 |  |  | 7.14% |
| **NO10**  **Margin** | *-* |  |  |  |  |  |  |
|  |  |  |  |  |  |  |  |
| **NO11**  **Tumor** | *EGFR* | 20 | p.M766delinsMASV (c.2296_2297insTGGCCAGCG) | chr7:55248998 | - | TGGCCAGCG | 15.58% |
|  | *FAT1* | 2 | p.E783K (c.G2347A) | chr4:187628635 | C | T | 7.91% |
|  | *SPRY4* | 3 | p.D238N (c.G712A) | chr5:141694031 | C | T | 3.41% |
| **NO11**  **Margin** | *-* |  |  |  |  |  |  |
|  |  |  |  |  |  |  |  |
| **NO12 Tumor** | *TP53* | 5 | p.P152L (c.C455T) | chr17:7578475 | G | A | 60.39% |
|  | *EGFR* | 19 | p.746_750del (c.2236_2250delGAATTAAGAGAAGCA) | chr7:55242466 | GAATTAAGAGAAGCA | - | 50.37% |
|  | *FANCE* | 5 | p.R371W (c.C1111T) | chr6:35426215 | C | T | 47.81% |
|  | *ARID1A* | 1 | p.G108D (c.G323A) | chr1:27023217 | G | A | 1.42% |
| **NO12**  **Margin** | *ARID1A* | 1 | p.G108D (c.G323A) | chr1:27023217 | G | A | 0.53% |
|  |  |  |  |  |  |  |  |
| **NO13**  **Tumor** | *EGFR* | 19 | p.746_750del (c.2236_2250delGAATTAAGAGAAGCA) | chr7:55242466 | GAATTAAGAGAAGCA | - | 61.48% |
| **NO13**  **Margin** | *-* |  |  |  |  |  |  |
|  |  |  |  |  |  |  |  |
| **NO14**  **Tumor** | *EGFR* | 19 | p.ELREATS746delinsA (c.2237_2254delAATTAAGAGAAGCAACAT) | chr7:55242467 | AATTAAGAGAAGCAACAT | - | 5.33% |
|  | *PKD1* | 43 | p.A3981V (c.C11942T) | chr16:2140946 | G | A | 1.42% |
|  | *MET* | 17 | p.1145_1161del (c.3435_3482delGGGAATCATCATGAAAGATTTTAGTCATCCCAATGTCCTCTCGCTCCT) | chr7:116418870 | GGGAATCATCATGAAAGATTTTAGTCATCCCAATGTCCTCTCGCTCCT | - | 0.74% |
| **NO14**  **Margin** | *-* |  |  |  |  |  |  |
